# Undisclosed probing into decision-making capacity: a dilemma in secondary care

**DOI:** 10.1186/s12910-021-00669-5

**Published:** 2021-07-23

**Authors:** Sandip Talukdar

**Affiliations:** 1 Cumbria, Northumberland, Tyne and Wear NHS Foundation Trust, Carleton Clinic, Cumwhinton Drive, Carlisle, Cumbria, CA1 3SX UK; 2 School of Social Sciences and Law, University of Manchester, Oxford Road, Manchester, M13 9PL UK

**Keywords:** Decision-making, Mental capacity, Liaison psychiatry, Probing, Psychiatrists

## Abstract

**Background:**

The assessment of patients’ decision-making capacity is ubiquitous in contemporary healthcare. This paper examines the ethics of undisclosed probing of capacity by psychiatrists. The discussion will refer to the law in England and Wales, though the highlighted issues are likely to be relevant in similar jurisdictions.

**Main text:**

Decision-making capacity is a private attribute, and patients may not necessarily be aware that one of their personal abilities is being explored. Routine exploration of capacity has not historically been a part of psychiatric examination, but it is now difficult to avoid during psychiatric interview.Ethical practice and shared decision-making require patients to be aware that their decision-making may be evaluated by the doctor at some point, and the potential implications of an objective professional conclusion of incapacity. Case law directs that patients should be informed about any assessment of their decision-making ability, though the extent to which this has translated into practice is unclear. However, explanation about the assessment may cause a patient to react negatively, which may impede therapeutic engagement and constitute an ethical dilemma. It is argued that in the absence of systemic measures, professionals should retain the discretion to decide whether a particular patient should be informed about the impending probe into their decision-making ability, or not. In the latter instance, concealment of information about the assessment or its purpose should be subject to the caveats and safeguards associated with any recourse to therapeutic exception.

**Conclusion:**

The necessity to mandatorily inform patients about assessment of their capacity introduces a novel ethical dilemma for psychiatrists. The negotiation of this dilemma should not be the prerogative of the clinician, and requires systemic initiatives to ensure universal awareness of patients about the possibility of their capacity being assessed during their journeys through healthcare systems.

## Background

This paper explores the ethics of capacity assessment in the context of healthcare, with a focus on the role of psychiatrists. The assessment of capacity in the absence of the patient’s awareness is an understudied area of clinical ethics. There is substantial literature on the practicalities of capacity assessment [1–3]. However, there is limited commentary on the ethical aspects of professional evaluation of capacity, when the purpose of the assessment as well as the fact that such an assessment would be performed, is unknown to the patient. The discussion in this paper will aim to attract further academic discussion on the subject. The paper will refer to the current capacity law in England and Wales to illustrate the ethical issues, though the latter are likely to be identical in similar jurisdictions.

The initial section of the paper will look into the status of capacity in the contemporary clinical context, its ethical connotations, and why it might attract probing. The discussion will refer to the underlying principles of the Mental Capacity Act 2005 in England and Wales [4] (subsequently referred to as MCA) and its test of capacity, which interprets capacity as time- and issue-specific [4, s2(1)]. The term ‘capacity’ will be used in a similar sense throughout this paper. The following section will consider the ethical aspects of capacity assessments and identify how undisclosed probing of capacity may breach privacy, trust, and case law. It will be argued that the practice presents a dilemma when doctors may need to assess capacity of patients without prior disclosure or explanation. This paper will refer to the professional work of psychiatrists to construct arguments, but the ethical issues are relevant for other clinicians who are required to assess capacity.

## Main text

### Capacity in healthcare: the ethical backdrop

Respect of autonomy is central to healthcare, which is translated into the concept of informed consent in many jurisdictions [5]. The principles of the MCA are underpinned by autonomy, with room for paternalism when the person is unable to make informed decisions. Mental capacity law in other jurisdictions, such as Northern Ireland [6, s1] and New Zealand [7, s5] adopt a similar approach. Capacity tends to be viewed as the gatekeeper to autonomy [8, p. 90], though the relationship between the two is complex, and has been variedly interpretated by the courts in England and Wales [9]. The premier status of autonomy in healthcare has attracted scrutiny, with authors such as Conly suggesting that coercive paternalism cannot be categorically abandoned in healthcare [10, pp. 1–7]. Manson and O’Neill have observed that the contemporary view of autonomy as a property of individuals in the ‘form of individual independence’ is distinct from Kant’s concept of the term [11, p. 18]. But despite criticisms, the importance accorded to patient consent is a counter against medical paternalism that used to be the prevalent value in medicine. Contemporary healthcare promotes individual choice and non-interference as the underlying values, and patients are expected to ‘participate’ in their treatment, instead of being ‘subjected’ to interventions.

During such participation, the patient is required to make one or more decisions based on provided information. Informed consent is centred on the principle that persons should be facilitated and supported to make decisions for themselves, though Maclean has observed that autonomy requires some capacity for rationality that goes beyond mere self-determination [12, p. 13]. The deemed association with rationality is debatable. An allusion to rationality may act as a conduit for questioning the patient’s choice when their decision is perceived as unwise, illogical, or associated with a likelihood of harm. Such questioning is justified on the principle of beneficence arising from the duty of care borne by all healthcare professionals. The result is a perceived need for establishing or rebutting the validity of the patient’s decision, which would require an exploration into their capacity.

There are additional ethical and philosophical issues around capacity and the categorisation of patients as capacitous or incapactious. Such categorisation can be problematic, and there are questions over viewing capacity as similar to other individual attributes of patients such as height and weight [13, p. 79]. Other implications of evaluating the patient’s decision include the conflict of their opinion with that of the socially acknowledged expert, the associated power dynamics, and difficulties in interpreting the reasoning process of another person [14, p. 237]. The conflict between the patient’s preferred choice and what the expert perceives as ideal may drive a detailed exploration into capacity across healthcare settings, with potential involvement of a psychiatrist in a proportion of instances. Since clinical professionals share responsibility for protecting the patient from harm, the capacity assessment may be unavoidable in certain situations. However, this paper focusses on the ethics of disclosure about a planned probe of their capacity to patients. The *need* underlying the probe is a different issue.

It is acknowledged that the specific need for an exploration into the patient’s decision-making and the validity of their consent (or refusal) would depend on the individual circumstances of the patient and the antecedent risks. However, there is a problem when such probing occurs as a component of the standard psychiatric interview. In order to understand the latter, it is necessary to inquire into the pathways through which a patient may come to have their capacity explored.

### Pathways to assessment of decision-making capacity

As a legally defined attribute, capacity refers to the ability to make specific decisions at the necessary time, and not to the patient’s ability to make decisions in general. The legal elements of capacity law in England and Wales are discussed later in the next section. However, it is possible to identify two pathways through which a patient’s decision-making capacity may attract and receive specific scrutiny.

The first pathway occurs in situations when there is reasonable doubt about the patient’s capacity to decide on a particular issue [15, p. 52]. Such doubts or impressions have moral relevance since their underlying aim is to protect the person from harm, with the realisation that protection may require interference [16, p. 230]. Psychiatrists may be requested to provide an opinion about capacity on one or more issue in complex cases, or act as a source of expertise for other clinicians. Psychiatric services are part of secondary care in the National Health Service (NHS) in the United Kingdom, and a referral from primary care clinician or another secondary care professional is usually necessary for a psychiatrist’s involvement with a patient. A common example of this pathway is that of patients seen by liaison psychiatrists at general hospitals.

The second pathway relates to situations when a person is seen by a mental healthcare professional irrespective of setting, when an objective impression about capacity may be required. It is necessary to mention the constituents of a psychiatric interview and recent recommendations from the regulatory agency in this regard.

At its core, psychiatric interviewing of a patient entails obtaining a clinical history and performance of a mental state examination, with the latter comprising observation and description of the patient’s mental state at that moment in time. It includes descriptions of the patient’s appearance, speech, actions, and thoughts during the interaction [17, p. 5]. However, contemporary guidance in England suggests that a psychiatric interview need to incorporate an objective impression of the patient’s capacity. This is reflected in the recommendation of the Care Quality Commission, (CQC), the independent regulator of health and social care in England, about when capacity should be assessed. In its guidance to care providers, including hospitals, the CQC mentions:

As a service provider, you assess people’s capacity to make decisions as part of their normal assessment and care planning arrangements, *whenever this is needed*. [18, p. 4]

The guidance further states:

Assessments of capacity must be made where there *may* be an ‘impairment of or disturbance in a person’s mind or brain’ affecting their ability to make particular decisions. [18, pp. 4–5]

If the above are considered together, it would appear unavoidable for a detailed clinical interview done by a psychiatrist (or another professional on behalf of a health or social care provider) to incorporate an objective impression of the patient’s decision-making capacity. The necessity to record such observations is recognised and highlighted in the policies of multiple mental health NHS Trusts in England and Wales [19–21]. The MCA policy of one NHS Trust mentions:

Although the starting point must always be a presumption of capacity, health professionals should always make an assessment of an individual’s capacity to make particular treatment or care related decisions and record the findings in the relevant professional records [22, p. 9].

The rationale would appear to be that psychiatrists are involved with patients who have conditions affecting the mind, and their patient population is more likely to have difficulties in making informed decisions. The CQC acknowledges that affliction with the clinical condition does not necessarily mean that the person lacks capacity [18, p. 5], but a validation of the capacity status is perceived necessary during the clinical encounter.

The CQC observes in separate guidance to their assessors of health and social care service that a capacity assessment is required where there is ‘clear reason for overturning the presumption of capacity’ and when there are ‘concerns’ about a person’s capacity to agree to treatment [23]. The origin of such concern is unclear, and leaves room for wide interpretation. It may be questioned as to how a ‘clear reason for overturning the presumption of capacity’ may develop *before* the capacity assessment has been performed, because only an objective evidence of incapacity revealed through such assessment might rebut the presumption of capacity.

It is difficult to align the above recommendation with CQC’s guidance to providers that a capacity assessment ‘must be made where there may be an impairment of or disturbance in the functioning of the mind or brain’ [18, pp. 4–5]. It would appear that the possibility that a patient might be afflicted by the latter would warrant an inquiry into their decision-making. There are ethical connotations to such practice if being labelled with a mental health diagnosis, or referred for a psychiatric assessment, would count on their own as sufficient reasons for an exploration into patients’ decision-making.

It is also interesting to note that a number of contemporary clinical texts have incorporated insight and capacity as part of the comprehensive mental state examination [24, 25, p. 17]. Mental capacity has not been previously viewed as a clinical sign or hallmark of psychopathology. The introduction of the MCA may have contributed to the increased attention to capacity during psychiatric assessments [26]. However, irrespective of the origins of this practice, it is difficult to find other examples in medicine where the clinician needs to record a legal attribute alongside the initial clinical findings. It is helpful to look at the elements of the capacity law in England and Wales to aid further discussion about the ethical aspects of capacity assessment.

### Mental capacity Act 2005 (MCA)

The MCA provides a legal framework for pertinent decisions to be made by others on behalf of a person lacking the mental capacity to do so [4, s2(1)]. Capacity is defined in the statute as decision-specific and time-specific, which implies the person should be able to make the particular decision when it is required [15, p. 42]. The ability of the person (referred to as ‘P’ in the Act) to make *other* decisions is irrelevant.

The principles of the MCA stipulate that P must be assumed to possess capacity unless it is established to the contrary, P is not to be treated as unable to make a decision unless all practicable steps to help him do so have been taken without success, and P must not be viewed as incapacitous merely because he makes an unwise decision [4, s1(2)–(4)]. The first three principles apply to all adults above the age of 16 and are congruent with respect for the patient’s autonomy.

Two other principles of the MCA are applicable on or after the conclusion of P’s incapacity. These latter principles are permissive of paternalistic actions for P’s welfare, with the stipulation that any action or decision taken or made for or on behalf of P must be in his best interests, and it must be considered if the purpose is achievable in a way that is less restrictive of P’s rights and freedom [4, s1(5)–(6)]. In addition, the decision-maker must permit P to participate in the decision-making and any act done for him [4, s4(4)]. It is also necessary to consider P’s past and present wishes, feelings, beliefs, and values as far as those can be reasonably ascertained during the determination of his best interests [4, s4(6)].

In order to come under the Act’s purview, including any paternalistic interventions, it is essential for P to have an impairment or disturbance in the functioning of the mind or brain. This element, referred to as ‘the diagnostic test’, has a wide remit and a formal ‘medical’ diagnosis is not essential. It does not matter whether the condition is permanent or temporary [4, s2(2)]. Lack of capacity cannot be established ‘merely’ by reference to the person’s age, appearance, or ‘a condition of his, or an aspect of his behaviour, which might lead others to make unjustified assumptions about his capacity’ [4, s2(3)]. The diagnostic test is complemented by the functional test of capacity, with the latter encompassing P’s ability to understand and retain the information provided, use or weigh it to arrive at a decision, and communicate the decision via any means [4, s3(1)]. The assessor has to judge the presence or absence of capacity on the balance of probabilities [4, s2(4)]. The assessor must be satisfied that the lack of capacity is a direct result of the particular condition of the mind or brain.

For addressing a request concerning capacity, the assessor would need to explore all three elements, including the test of capacity, the diagnostic status, and the clinical condition being responsible for lack of capacity. For the psychiatrist, the process may involve making a new diagnosis that had not existed before or confirming a previous mental health condition relevant to the question about capacity. These are issues with ethical relevance but distinct from those concerning the undisclosed probe into capacity and are outside the scope of this paper.

### Necessary versus ‘unwarranted’ probe into capacity

The above discussion has highlighted that capacity assessment is now an essential aspect of the work of psychiatrists, who may need to explore the decision-making of patients referred to them by clinicians in other specialties, as well as during any detailed psychiatric interview irrespective of the context. It is suggested that there is a difference between ‘undisclosed’ or ‘unexplained’ assessment of capacity, and ‘unwarranted’ probe into a person’s decision-making.

Capacity assessment may be necessary in many clinical situations for a range of reasons, such as the need to confirm that a patient with specific needs has correctly comprehended the risks and benefits of an intervention. Due regard and adherence to principles of the MCA would ensure that actions congruent with beneficence are not undertaken unless there is confirmation that the patient lacks capacity, which is a safeguard against medical paternalism. The fourth and principles of the MCA are permissive of beneficent actions, applicable only after establishing that P is incapactious.

It is interesting to compare the above with situations where a psychiatrist interviews a patient, and the capacity assessment is not based on the evident needs of the latter but the fact that they are more likely to have a condition affecting their mind. There is a problem if mere referral to a psychiatrist, or the existence of a prior mental health diagnosis, are looked upon as sufficient reasons in themselves for probing a patient’s capacity. It would be stigmatising if the decision to probe turns on the patient’s diagnostic status alone. Unless the patient is aware of the probe into their decision-making, there is potential for the patient’s decision-making authority being unjustly violated, which remains unchanged even if the patient is found capacitous after the assessment.

Therefore, it is suggested that the non-awareness of the patient about the probe into their capacity would transform an assessment into ‘unwarranted’, though it may still be perceived as ‘necessary’ by the professional. The intertwined issues of violation of autonomy, stigmatisation, and breach of privacy acquire relevance when the assessment and its purpose are unknown to the patient.^1^ Among these issues, the breach of privacy deserves further consideration on account of its intimate association with autonomy and related rights.

### Undisclosed probing: breach of privacy

Definitions of the concept of privacy have proved challenging [27, p. 445]. Moore observes that the common element in all definitions of privacy is the exercise of control over one’s personal information [28, p. 216]. Parker describes privacy as control over when and by whom the various parts of us (as identifiable persons) can be sensed by others [29, pp. 283–284]. Moore further clarifies the definition of privacy as a right to exert a ‘certain level of control over the inner spheres of personal information and access to one’s body, capacities, and powers’ [28, p. 218]. These are engaged when a probe into capacity occurs in the absence of the patients’ awareness.

Capacity depends on the functioning of multiple aspects of the mind. These include the abilities to contemplate future possibilities and perform logical deduction. The individual’s personality, life experiences, and values may further contribute to the decision or choice. These are individual and private elements over which the person may justifiably wish to exert control. The person may knowingly and voluntarily waive this control under specific, defined, and regulated situations while being aware of the consequences, such as during formal examinations or job interviews. As an extension of this point, the capacity to make specific decisions would qualify as an aspect of an individual’s private life. The exposition of this attribute to inspection and judgment by others needs to remain a prerogative of the person.

The above point needs further discussion. Opinions, perspectives, and judgments made by humans in the societal context are open to scrutiny and criticism from others and have been so since historical times. Such opinions and reactions, which can be of a negative or disparaging nature, may need to be accepted as an unavoidable aspect of life in contemporary societies. However, professionals’ opinions are powerful and influential, and doctors’ opinions can have profound effects even outside clinical contexts.^2^ It is necessary for professionals to be discrete in applying their expertise. Arguments that one’s decisions and actions are always open to scrutiny in contemporary society are not extendable to situations when acknowledged experts carry out such scrutiny without the subject’s awareness.

It may be questioned whether the usual rights to privacy should continue to apply in clinical settings, since there is no objection to doctors performing physical examination during clinical encounters without detailed explanations at every step. However, the latter does not bear an equal comparison with the probing of capacity. Doctors may perform physical examinations to elicit symptoms and signs that would contribute to the overall diagnosis, and patients are aware that the clinician is making certain observations about their body and state of health.

However, decision-making capacity is distinct from ‘symptoms of the mind’, or psychopathological features identified during the psychiatric examination that contribute to diagnosis and formulation. Judging a patient as incapacitated is not comparable to identifying a clinical sign that contributes to identifying a particular illness. The patient may not necessarily be aware that a judgement has been made about one of their personal attributes.

Patients do not automatically discard all of their privacy-related rights during interactions with healthcare professionals. Hospital admission and clinical examination require temporary waiving of privacy on the basis of valid consent, unless the patient happens to object and interventions are required to protect them or others. In mental health settings, the use of specific statutes such as the Mental Health Act in England and Wales may be necessary for the purpose. Respect for privacy and autonomy warrants that the patient’s consent to being interviewed should not be construed as a licence to probe into decision-making, particularly when the specific probing may have repercussions on their rights. Respect for patient privacy is associated with the dignity that the patient deserves in healthcare settings. Statutory regulations in England stipulate that service users must be treated with dignity and respect [32, Regulation 10]. Dignity is a complex concept [33], but it bears a close relationship with privacy and freedom of choice. The latter are fundamental to respect for autonomy.

### Breach of trust

Multiple authors hold trust to be the cornerstone of the doctor-patient relationship [34–36]. Trust in the doctor-patient relationship relates to the broader framework instead of the professional, with trust on the institute or healthcare system being the significant issue [35, pp. 67–68]. However, as pointed out by previous writers such as Baier [37] and Hawley [38], and more recently summarised by Nickel and Frank [39], trust can also serve as a ground for exploitation and may not necessarily have beneficial connotations.

The relevance of institutional trust is evident when patients engage with psychiatrists under the impression that the specialist would be able to help them, which is reflective of their trust in the healthcare system and the medical identity of the professional.^3^ Unless the patient has prior information about the purpose of the evaluation and potential consequences,^4^ active probing for information that is private and bears legal relevance would be problematic from multiple angles, including future use of such information.

Patients have traditionally had little safeguard against the probing and recording of personal information,^5^ apart from isolated exceptions such as testing for HIV status.^6^ In general, it has been the traditional practice for doctors to examine any aspect of the patient’s body and life situation if it is considered relevant to wellbeing and recovery. In physical medicine, such probing may extend to the patient’s private body regions or aspects of his personal life, including sexual relationships. Psychiatrists may question and probe aspects of a patient’s personal history that lacks a direct connection with health but might be associated with potential risk.^7^ Capacity assessment may centre on discovering whether the patient’s decision incorporates their correct comprehension and appreciation of risks. However, collaborative working with patients requires them to know the reason for such questioning and why it might be pertinent.

### The professional, the probing, and the aftermath

The psychiatrist’s values and attitudes are other aspects that require consideration in the discussion of the ethics of this practice. Buchanan is of the opinion that a psychiatrist’s opinion about capacity invariably possesses moral connotations and is never purely clinical [42, p. 353]. Such connotations could be influential when the patient’s capacity is interpreted as ‘questionable’ or ‘fluctuating’ [43, p. 198]. Banner has suggested that doctors have their own perspectives about how patients ‘ought to’ appreciate the information while making decisions [44, p. 1040], and her argument has received support from the psychiatric profession [45]. Banner also observes that capacity assessments are essentially normative judgments of the assessors [44, p. 1040]. Keene is forthright in his view that mental capacity is ‘in the eye of the beholder’, and there is a need to focus more on the values and pre-conceptions of the assessor rather than the person whose decision-making is under scrutiny [46].

The above opinions need consideration with the fact that the history of psychiatry has, for the most part, centred on paternalism, where patients were for the most part ignorant about the interventions carried out in their (supposed) best interests. This aspect of the specialty’s history requires viewing in conjunction with the societal expectation to protect the mentally unwell from their harmful decisions and actions. Empirical studies have found a ‘deep but largely unrecognised difference in values’ between psychiatrists and other professionals involved with persons who have mental health needs [47, p. 67]. Although case law stipulates that assessors of capacity (including psychiatrists) must not act on the ‘protection imperative’ [48, para 65], it remains unclear as to whether psychiatrists are able to jettison their traditional role and values that require them to protect the patient from harm.

For example, it is helpful to look back to patients at general hospitals who may be referred to liaison psychiatrists. It is difficult to come across reliable figures on how often such patients are aware of the liaison psychiatrist’s role, or that a psychiatry specialist would assess them. The lack of figures is understandable since a fair proportion of patients may have impaired cognition or decreased sensorium.^8^ In addition, a number of patients may harbour apprehensions or misconceptions about psychiatrists, reinforced by the stigma that persists against the specialty and its practitioners. However, it is possible to distinguish the involvement of the psychiatrist from that of other medical specialists.

A psychiatrist’s involvement can lead the patient down a different path that has connotations to her rights. For example, a new psychiatric diagnosis may come to exist for some patients, with repercussions that might not be immediately evident. In contrast to discussions over adverse effects of medical intervention, there is no requirement for the patient to be informed about the possible *aftermath* of a capacity assessment.^9^ This is understandable from a practical perspective but raises questions about whether current practice amounts to a limitation of the patient’s autonomy at the onset of the referral to the psychiatrist. A patient who is unaware that an assessment of their decision-making capacity has occurred and the professional has found them incapacitous, would have no evident means to challenge the decision, unless supported by other agencies such as families and advocates. It is not difficult to see how a practice of undisclosed capacity assessment may contribute to persistent suppression of patient autonomy in such circumstances.

The assessment of a personal attribute requires viewing together with respect for the person. Harris identifies two essential elements to ‘respect for persons’, which comprise concern for the said person’s welfare and respect for his/her wishes [49, p. 193]. It is unclear if the second element is consistently fulfilled during psychiatrists’ assessments of capacity. The power differential between the patient and the professional is an additional exacerbating issue since the patient lacks the opportunity to express their preference about the procedure. The implicating factors include the act of probing a private attribute, the consequences being uncertain or unknown to the patient, a likelihood for some patients being labelled as incapactious or incompetent, and practical hurdles that the patient might face in seeking a review of the opinion.

The consequence of being labelled as ‘incompetent’ can be severe, as pointed out by Faden and Beauchamp:

If the label of “incompetent” is placed on a patient or a subject, a train of coercive events is potentially set in motion. The label “competence” commonly functions to denote persons whose consents, refusals, and statements of preference will be accepted as binding, while “incompetence” denotes those who are to be placed under the care and control of another’ [50, p. 290].

Although the above observations were made in the North American context, the situation is not appreciably different in England and Wales if ‘incompetent’ is substituted with ‘incapacitated’. Being judged as incapacitous has the potential to determine the patient’s future in a manner akin to diagnostic labelling. Thus, there are grounds to view the practice as an unwarranted exploration or interference with the patient’s private life.

In the context of the general hospital, it is the initial impressions of non-psychiatrists that may herald further scrutiny from psychiatrists. During the assessment, the patient lacks any apparent means to counter such scrutiny, apart from disengagement or expressing a request to be left alone [51]. Such refusal or reluctance would reinforce any pre-existing doubt over the patient’s capacity.

The professional’s probing bears the potential for disempowerment with an impression of incapacity. Moral aspects of the exploration into another person’s decision-making transcend the interaction between the two parties, one reason for which is the limited consideration to relational aspects of decision-making, as pointed out by Camillia Kong [52, p. 2]. This point needs consideration alongside the expectations harboured by the patient, their family, and the healthcare or social systems, which might not necessarily align. Contributors to this incongruence of expectations include the perception of psychiatrists as agents of control [53] and the ‘social contract’ between psychiatry and society [54].

### Breach of regulations and case law

Information obtained and recorded during a psychiatric assessment would constitute data, with laws governing its use. The relevant statute in the UK is the Data Protection Act 2018 [55]. Information concerning a person’s decision-making capacity would be considered sensitive data owing to its direct association with mental health. The patient has additional rights concerning the data as declared in the NHS Constitution [56]. These include the right to receive information about treatment options along with their risks and benefits, access to one’s healthcare record and correct of factual inaccuracies, confidentiality, and being informed as to how the information might be used [56, 3(a)].

It is worth considering the above patient rights with the assessment of decision-making capacity. The Code of Practice to the MCA states that the professional should ‘Make every effort to communicate with the person to explain what is happening’ [15, p. 56] but with little further elaboration. It is, therefore, up to the individual clinician to decide the extent of disclosure. However, given the potentially severe aftermath of capacity assessments for patients, including the likelihood that the power to make certain decisions could be taken out of their hands, there is a need for official guidelines to incorporate stringent specifications regarding explanations and sharing of information. In the absence of such explanation, the collected information and resultant opinions on capacity would amount to an unwarranted exploration of the person’s private life and cognitive processes. There may be a role for psychiatrists in facilitating conversation and communication, but their involvement as an external professional should be based on mutual agreement with the patient.

An additional issue is subsequent sharing of such data or clinical opinion suggestive of the patient’s capacity status with other clinicians and its use to judge the patient’s decision-making on subsequent occasions, which would be problematic and incongruent with the law. A physician who forms an opinion based on a previously noted psychiatric opinion about incapacity would contribute to unfair stigmatisation of the patient, even if it is established that the patient’s circumstances have not undergone significant change between the two occasions.^10^

Doctors have traditionally counted on beneficence to defend intrusions on the person. However, the obligatory examination of capacity in psychiatry presents a singular breach of autonomy-related rights and privacy. Assessors of capacity need to be aware of the observation made by Hayden J in *Wandsworth* about the examination of capacity. The case concerned care proceedings of three boys, the eldest of whom was over 16, and there were questions about his capacity to decide on where he should reside. It was observed.

It seems to me that a prerequisite to evaluation of a person's capacity on any specific issue is at very least that they have explained to them the purpose and extent of the assessment itself. Here, that did not happen. In my view, it is probably fatal to any conclusion. In any event, it, at least, gravely undermines it [57 at 49].

The above point was reinforced in the more recent case of *DP v Hillingdon*, where Hayden J observed that an omission in explaining the reason for the assessment to P could raise doubts about whether the assessor had fully grasped the reason underlying the assessment and the question that needed answering [58 at 18–19]. The observation of Parker J in *PC v City of York* remains pertinent in this context:

… there is a space between an unwise decision and one which an individual does not have the mental capacity to take and … it is important to respect that space, and to ensure that it is preserved, for it is within that space that an individual's autonomy operates [59 at 54].

There are far-reaching implications to these observations, and it reinforces the necessity for patients to be aware of the purpose and extent of the assessment of capacity. The identification and respect of the ‘space’ referred to by Parker J includes clarity on the exact issue that P needs to decide. The issue is central to patient autonomy. However, it introduces additional questions about whether an isolated explanation about the impending probe into decision-making is sufficient for respect of P’s autonomy.

The examination of decision-making should require P to be aware of the professional’s identity, the reason for the latter’s involvement, an outline of the assessment process, and the implications of the opinion. These would appear extensive, daunting, and potentially overwhelming for the patient. The issue requires viewing in the context of controversies that embroiled psychiatry in the past [60]. It is necessary to ensure that probing and recording of capacity does not result in further unjustified labelling, with ‘incapacitated’ coming to attain a place alongside historical adjectives like ‘insane’, ‘mad’, or ‘lunatic’.

The above assertion may invite the response that capacity assessment is not overly complicated in many situations, and it is often possible to summarise it in a single sentence stating there is no current concern about the patient’s capacity. From a practical perspective, this might be a workable option for laypersons and non-psychiatry clinicians. However, blanket statements have little utility in psychiatry or law in the absence of clearly articulated reason or evidence to the opinion [61, para 20]. Another flaw with the above suggestion is the assumption that most patients should have no objection to a doctor recording an impression about their capacity during a clinical encounter. Such assumptions are speculative since it is difficult to predict how an individual patient might interpret the process. The extent of the inquiry and the fact that capacity is a legal construct makes it necessary to reinforce the requirement for the patient to have sufficient awareness of the process, including the fact that observations and impressions are being made and recorded in the patient’s clinical files and may be referred to in the future by other professionals.

#### The dilemma for the professional

The above discussion has highlighted issues with the routine probing into capacity as part of psychiatric assessments. The observation in *Wandsworth* makes it difficult for professionals to avoid telling patients that their decision-making capacity would be evaluated. There is a concomitant necessity to consider the effects of the disclosure on the patient. The patient’s reaction would depend on realising that one of their abilities was being questioned, and interpreting it as an ego threat [62]. There are multiple definitions of ego threat, though the common element is a threat to the person’s self-image or self-esteem [62, p. 154].

The self-image of a person includes their belief or confidence about their abilities in deciding and performing activities. Several factors contribute to a person’s sense of self-esteem, including how they might appraise their performance, abilities, and interactions with others [63]. For an adult, being informed that their abilities are under question is hurtful and threatening to their self-image and self-esteem. The reaction to such a threat may be cognitive, behavioural, or emotional [62, p. 151]. A patient who comprehends the disclosure would have reason to interpret that they were being treated differently from other patients, and such a realisation may be dissonant with their previous experiences during interactions with healthcare professionals. The patient’s impression of being discriminated against in some form, or their abilities being rejected at the onset, are further possibilities. Under such circumstances, a person’s usual response would include negative affect, reduced self-esteem, and other constructions of the rejection experience [64].

The negative reactions may manifest in anger or verbal aggression that are known responses to ego threat [65]. Disengagement with the interviewer and refusal to continue with the assessment are possibilities for a proportion of patients. However, such disengagement may lead to persistence or reinforcement of the doubt about their capacity, and re-establishment of the therapeutic relationship with the professional may also be difficult.

Another issue with ethical implications is the breach of trust that may occur. Some patients may be able to understand that the psychiatrist is aiming to gain an understanding of their decision-making capacity without explaining the reason to them. In addition, there would be a possibility that the patient could learn about the probe into their capacity and the professional conclusion at a subsequent time. Even if the psychiatrist had concluded that there was no doubt over capacity, it would be an insufficient counter against the patient’s realisation that one of their abilities had been judged without their awareness.

It is helpful at this juncture to summarise the above points. The clinical situation involves a psychiatrist interviewing a patient. The patient may not be necessarily aware that a psychiatric opinion had been sought,^11^ which raises distinct ethical issues on its own, but is not the main topic of discussion in this paper. The psychiatrist needs to record their impression about capacity as part of a comprehensive psychiatric assessment which, at the minimum, should be the patient’s capacity to consent to ongoing care and interventions.^12^ The patient is unaware that the psychiatrist would evaluate their decision-making capacity on one or more issues during the encounter.

The patient needs to have relevant information for making decisions, and the fact that their decision-making capacity was going to be examined (and concluded upon) would rank among the relevant information. For convenience, this may be termed disclosure about impending assessment of capacity. For patients subjected to  probe of capacity, withholding such information would constitute a breach of privacy, trust, relevant regulations, and case law. There could be an inherent circularity to the claim that disclosure about impending assessment of capacity is a separate requirement since the need for such disclosure is inherent to the patient’s requirement for information pertinent to the decision that needs to be made.^13^

It is suggested that the disclosure about the impending assessment of capacity is not ‘inherent’ to disclosure of the primary main information pertinent to the specific issue that the patient must decide on. Let us consider a patient who has presented at casualty and needs to decide between accepting treatment at home or being admitted to hospital for the management of a physical health condition. Let us assume that this patient also has a mental health condition, with difficulty in weighing or balancing information as a consequence. The relevant information for any patient would comprise the relative advantages and disadvantages of being admitted to the hospital, which would entail accepting some restriction of liberty in exchange for supervised care and possible earlier recovery. The disclosure about an impending assessment of capacity is ancillary or tangential, and not ‘inherent’, since the former depends on an attribute of the patient who has an identifiable mental health condition. It is distinct from the core information that the patient needs to make a choice—which in this case would involve the advantages and disadvantages of hospital admission and care at home. Disclosure about impending assessment of capacity would be irrelevant for patients who have the same underlying physical health condition but do not have any mental health need.

Disclosure about an impending assessment of capacity is congruent with the transparency expected in doctor-patient relationships but is associated with a risk of the patient reacting negatively and withdrawing from the engagement. The latter could constitute greater harm. In the practical world, it could be challenging to predict the reaction of an individual patient, and the psychiatrist would need to draw upon their expertise, ancillary information, and objective impression during the initial stages of the interview to estimate the effect of disclosure on the patient. In addition, there is a moral dilemma since the disengagement of the patient may reinforce the doubt about their capacity with a possibility of further coercive measures in the future.

The situation where the professional foresees a patient reacting negatively to disclosure about the impending capacity assessment gives rise to an interesting consideration. Is it possible that the disclosure may impact a particular patient in a manner that they lose the ability to make an informed and reasoned choice? The suggestion might appear speculative and unusual, but there is no reason as to why a particular person’s emotional reaction could not disrupt their reasoning faculties on learning about a threat to their ego and self-image, with their decision-making capacity under question from a professional hitherto unknown to them. Decision-making is susceptible to disruption by emotional reactions [66]. Cave has argued that persons who are severely impacted by certain information during decision-making are incapacitous, and the offending information may be withheld in their best interests under the MCA [67]. The question is whether the same analogy is applicable to non-disclosure of the information about the impending probe into their capacity.

At first sight, it is difficult to dismiss Cave’s proposition. However, disclosure about a planned capacity assessment stands out from other information that the patient may to make a particular decision. First, the disclosure about an impending assessment of capacity is not part of the information concerning the specific clinical or social issue requiring a decision from the patient, as mentioned earlier. Second, there is a legally recognised need for this disclosure at the onset. Third, the doctor may believe that the disclosure would significantly impact the patient, but such an impression would not be based on objective evidence – since no disclosure is actually made, and the anticipated reaction of the patient could be seen as ‘predicted’ or based on surmise. Finally, in the absence of disclosure, it is possible that a patient may be able to guess the purpose of the interview and the ongoing probe into their decision-making capacity, with a resultant breach in trust. Cave’s suggestion, therefore, may require caution when applied to the disclosure of impending assessment of capacity and its purpose.

There is the possibility that the approach of the psychiatrist and the use of skilful language could reduce the likelihood of patient disengagement. There is a difference between the patient being informed that their decision-making capacity would be assessed and telling them that the doctor would ask some further questions to confirm that they had understood all relevant information for making their own decision. The latter option would sound less confrontational on its face, though there is no difference in the underlying purpose of the questioning.

However, there is no guarantee that the use of appropriate ‘patient-friendly’ language and communication skills of the psychiatrist would address all situations where there is a potential for the patient to react negatively, on learning that the assessor harbours doubt about their capacity to make decisions. Irrespective of the language that the psychiatrist might use during the explanation, the resultant opinion would still be recorded under the heading of capacity in the clinical records. Although the friendlier language may help avoid a practical dilemma, there are moral connotations if the patient remains unaware about the evaluation of the legal attribute and the repercussions it might have for them.

It will be a morally troubling situation if the psychiatrist develops the impression that disclosing the purpose of the questioning would result in a negative outcome for the patient, and non-disclosure would be a helpful way to circumvent the dilemma. Nevertheless, it is now hard for professionals to sidestep the dilemma in its entirety. If the psychiatrist perceives the risk of disengagement to be significant, which might lead to further measures of hard paternalism, then limited withholding of information would be justified. It is suggested that non-disclosure should attract restrictions similar to those on therapeutic exception (TE).

### Viewing non-disclosure as similar to therapeutic exception

The medical profession has a long history of doctors hiding the truth from their patients [68]. However, it is worth noting that non-disclosure in manners similar to TE is contrary to ethical principles in professions other than medicine [69, p. 624]. Cox and Fritz have argued that intentional non-disclosure by any means, including withholding information, is morally equivalent to lying in the doctor-patient relationship [70]. The necessity to disclose the purpose of capacity assessment requires viewing in a similar perspective. Higgs suggests that TE is driven by doctors attempting to protect themselves from ‘the pain of telling truths’, which benefits them more than their patients [69, p. 624]. An intent to avoid a morally troubling situation echoes Higgs’s description of doctors wanting to avoid the ‘pains of telling the truth’ to the patient [69, p. 624]. There are thus reasons to view non-disclosure of the aims of a capacity assessment similarly to TE.

The judgment in *Montgomery* introduced stringent restrictions on TE [71 at 91], which were reinforced and further limited by the GMC in its 2020 guidance towards doctors [72]. At present, a time-limited recourse to TE remains available to doctors in the UK, who must seek legal advice if any information requires withholding for a sustained or unclear length of time [72] para 14–15]. For moral defensibility, TE needs to be ‘patient-centred’ in the contemporary sense of the term. There could be justification for TE when full disclosure would ‘seriously compromise the patient’s permissible ends’, and any non-disclosure would not conflict with other interests of the patient that are morally important [73, pp. 333–34].

TE relates to information about the diagnosis, prognosis, and material risks of medical intervention. It is unclear whether withholding information about an impending assessment into the patient’s capacity would fit into any of these categories. However, it is logical to suggest that important information relevant to the assessment should attract safeguards similar to those governing patients’ access to information about diagnosis and treatment. There remains scope for further debate on this point, but the option would offer a defence for the professional if they can demonstrate that a deviation from the legal recommendation was necessary to avoid anticipated harm that would have caused more profound effects upon the patient.

Non-disclosure of the purpose of capacity assessment may have certain similarities with the withholding of information by doctors in other aspects of clinical encounters. However, there are doubts as to whether indefinite delaying of such disclosure would be possible since disclosure would need to occur at some point according to the recent guidance of the GMC. The resultant effect on the patient would be similar: namely that one of their innate attributes had been examined without their awareness, which would leave scope for adverse reaction on the part of the patient. It would also not address the problem arising from the possibility of the patient realising the purpose of the questions probing their capacity during the interaction. There is, therefore, no assurance that non-disclosure on grounds similar to TE would address professionals’ moral dilemma.

### Looking at ‘universal’ disclosure

The alternative to doctors disclosing the aim and purpose of the capacity assessment to individual patients could take the form of patients receiving information at the onset of their contact with the healthcare services that their decision-making might be checked on one or more occasions during clinical encounters. It would be necessary for the purpose to be explained to the patients: that it is a legal necessity for clinicians to explain the risks associated with their clinical condition and proposed interventions, and an assessment of this sort was necessary to ensure that patients did not unknowingly shoulder any risk without appreciating its magnitude. Information of this nature could be made available in various ways, including inclusion in the clinic appointment letters, mobile phone text messages, email confirmations of appointments, or patient portals of organisation websites.

Such initiatives may still fall short of the target to ensure awareness of all patients about the possible examination of their decision-making capacity.

For example, the provision of the information would not necessarily result in the patient reading and comprehending them. Patient literacy is a significant issue, and not all patients may have the literary skills to comprehend the information [74, 75]. Research has demonstrated the gap between the patients’ informational needs and the information they receive in the hospital [76]. Empirical findings from the North American context reveal that patient portals may fail to educate patients properly [77]. There is no guarantee that universal prior intimation would ensure an individual patient being aware of the exploration into their decision-making.

However, there would be room for the incorporation of population-based provision of information with individual or one-to-one explanations during the actual clinical encounter. Questions would remain as to whether these would be sufficient for certain patient subgroups. Examples would include persons with intellectual difficulties and patients presenting with a clinical emergency who may subsequently be assessed by liaison psychiatrists. It is surmised that it should not be a major hurdle to identify and implement innovative solutions to such challenges. However, the prerequisite to any solution would be recognising the necessity for patients to be aware of the potential exploration of their capacity during healthcare encounters.

In summary, the need to inform patients about an impending assessment of their capacity presents a significant ethical dilemma. There are suggestions in literature that professional opinions about competence or decision-making capacity should be viewed as matters of ethical judgment that need not rigidly adhere to notions of autonomy [78]. However, such notions need viewing alongside the consideration of patients’ rights, where the latter are reinforced by regulations, case law, and professional guidance.

Non-disclosure of the purpose of a capacity assessment poses the risk of the patient remaining unaware of the outcome, which would be a barrier to the assertion of their rights, such as the right to seek a second opinion. A combination of candour on the part of the healthcare services and individual professionals in explaining the reason for any exploration of decision-making capacity to patients may be one way of addressing the dilemma. However, this would be difficult to pursue in the absence of recognition of the problem.

## Conclusion

This paper has highlighted and questioned the prevalent practice of capacity assessment in the absence of disclosure. Decision-making capacity is morally distinct from doctors’ assessment of physical and mental functions to aid diagnosis. There are ethical implications of patients being unaware of such probing and its repercussions, including the potential uses of the obtained information. Non-disclosure facilitates the labelling of patients as incapacitous without their awareness, leaving them powerless to challenge the professional opinion unless supported by other agencies such as families and advocates. The lack of awareness may result in a breach of patients’ trust on professionals and the healthcare system. A stipulated need to record a patient’s capacity to consent to the interview and interventions in mental health settings leaves room for stigmatisation. The same may occur with a professional opinion of incapacity being cascaded and applied by other clinicians in different contexts.

Case law has recognised the necessity to inform patients that their decision-making capacity may be examined, but it is unclear if the stipulation has translated into formal policies. However, the judgment in *Wandsworth* has accentuated the dilemma for the professional. The stipulation that patients must be aware of the probing into their capacity would need to balance the harm caused by the reaction on the patient’s part. Measures to avoid such harm through non-disclosure would constitute paternalism.

The disclosure about an impending assessment of capacity may present an ethical dilemma for professionals in certain clinical contexts. The balancing of harms may require professionals to withhold information about the probe into the patient’s decision-making. This paper has suggested that such withholding should attract restrictions similar to those imposed on TE at the least. The current situation is unsatisfactory, and future options may need to incorporate broader messaging to patients and the public about this aspect of doctor-patient encounter in healthcare. However, recognition of this dilemma is an essential prerequisite for the multifaceted approach necessary to ensure patients are party to the decisions made about them.

## Data Availability

Not applicable. Does not include data, and no datasets were generated or analysed during the work on this paper, or the PhD project that generated this paper. All references have been cited, with doi numbers or website links wherever possible.

## Abbreviations

CLP: Consultation-liaison psychiatry
CQC: Care Quality Commission
MCA: Mental Capacity Act 2005
NHS: National Health Service
UK: United Kingdom

## Footnotes

1 I would like to thank one of the anonymous reviewers for highlighting this to me.

2 The case of Meadow, who was struck off the medical register in the UK on account of giving ‘erroneous and misleading statistical evidence’ [30] is an example, though the courts subsequently ruled in his favour [31].

3 This refers to patients who are able to engage with the psychiatrists. It should be noted that the opposite may also occur in psychiatry. Patients with past experience of involvement with psychiatry that they found distressing or traumatic may view the psychiatrist with distrust and suspicion, which would be a barrier to the establishment of rapport and engagement. But such a situation would also exemplify distrust directed at the institution rather than the particular professional.

4 This could occur, for example, with ‘experienced’ patients who happened to undergo a similar assessment before but later came to know about the opinion formed by the psychiatrist.

5 Prior to the Bristol heart scandal in the 1990s, there were little safeguards to the uses to which any medically obtained information could be put to [40, p. 3].

6 However, recent guidelines suggest a formal written consent is no longer necessary for universal HIV testing of patients, as long as they understand that they are being tested for HIV [41, p. 2].

7 For example, a psychiatrist assessing an elderly male farmer with depression living on his own in an isolated area, would most likely ask a screening question of whether the patient has possession of any firearms at his dwelling.

8 This is another area that requires empirical investigation.

9 The aftermath could include the patient being viewed as incapactious and decision-making being removed from the patient at least temporarily. Unless the patient is informed about the conclusion about their capacity, it could be difficult for them to challenge the opinion of the psychiatrist.

10 I would like to thank one of the anonymous reviewers for highlighting this.

11 This situation may arise in general hospitals where a referral may have been made to a liaison psychiatrist.

12 It is possible that other specific capacity assessments may be required. One example would be the capacity of an elderly inpatient to decide between being discharged to their own home or a care home.

13 I would like to thank one of the anonymous reviewers for pointing this out.
